# 519. Early Transition to Outpatient Remdesivir Increases Patient Satisfaction while Decreasing Hospital Costs

**DOI:** 10.1093/ofid/ofad500.588

**Published:** 2023-11-27

**Authors:** Supavit Chesdachai, Christina G Rivera (O'Connor), Jordan K Rosedahl, Lindsey Philpot, Ruchita Dholakia, Bijan J Borah, Evan Draper, Richard Arndt, Ravindra Ganesh, Jennifer J Larsen, Molly J Destro Borgen, Raymund R Razonable

**Affiliations:** Mayo Clinic, Rochester, MN; Mayo Clinic, Rochester, MN; Mayo Clinic, Rochester, MN; Mayo Clinic, Rochester, MN; Mayo Clinic, Rochester, MN; Mayo Clinic, Rochester, MN; Mayo Clinic, Rochester, MN; Mayo Clinic, Rochester, MN; Mayo Clinic, Rochester, MN; Mayo Clinic, Rochester, MN; Mayo Clinic, Rochester, MN; Mayo Clinic, Rochester, MN

## Abstract

**Background:**

Remdesivir is FDA-approved for the treatment of hospitalized patients with severe COVID-19. Many patients improve clinically to allow for hospital dismissal before completing the 5-day course. In prior work, those who continued remdesivir in an outpatient setting had better outcomes. Here, we assessed patients’ perspectives and the economic impact of this outpatient practice.

**Methods:**

During a one-year period, 3,029 patients received remdesivir for COVID-19 in the hospital; of them, 542 were dismissed to continue remdesivir in the outpatient setting and were surveyed. The cost of care was compared between those who remained in the hospital vs. those who were dismissed.

**Results:**

470 of 542 patients were surveyed, and 93 (19.8%) responded. Responders were older than non-responders (median, 63 vs. 58 years). The majority (70.5%) had symptoms resolved by the time of the survey. Ten patients (11.4%) had persistent symptoms attributed to long COVID-19, including fatigue (30.1%), brain fog (23.7%), and insomnia (18.3%). 73.8% were excited to learn about the outpatient remdesivir program. The majority (81.0%) felt that receiving therapy at home was the right decision; 79.5% had support and felt the benefits of completing treatment at home. The majority were satisfied with communication before (75.6%) and during (80.5%) the outpatient appointment, information on benefits (80.3%) and risks (58.5%), time spent at the infusion center (88.6%), quality of care (82.3%) and overall experience (76.0%). The benefits of treatment were high-value (82.6%), while the infusions' cost, inconvenience, and discomfort were unimportant for most responders. After adjusting for gender, comorbidity score, and WHO scale, the predicted costs for the groups were $16,544 (inpatient) and $9,097 (outpatient) per patient (difference of $7,447; p< .01). During this study, we estimated a total of 1,077 hospital room-days were made available to other patients.

**Conclusion:**

An outpatient remdesivir program that allowed for early dismissal was perceived favorably by patients hospitalized for COVID-19. The program resulted in significant cost and resource savings, the latter in terms of the availability of hospital rooms for other patients needing critical services.

**Disclosures:**

**Christina G. Rivera (O'Connor), Pharm.D**, Gilead Sciences: Advisor/Consultant|Gilead Sciences: Board Member|Gilead Sciences: Grant/Research Support|Gilead Sciences: Honoraria **Bijan J. Borah, Ph.D.**, Boehringer Ingelheim: Advisor/Consultant|Exact Sciences: Advisor/Consultant **Ravindra Ganesh, M.B.B.S., M.D.**, Alpaca Health: Advisor/Consultant **Raymund R. Razonable, MD**, Allovir: Endpoint Adjudication Committee|American Society of Transplantation: Board Member|Gilead: Grant/Research Support|Novartis: DSMB|Regeneron: Grant/Research Support|Roche: Grant/Research Support

